# Patient related outcomes in a real life prospective follow up study: Allergen immunotherapy increase quality of life and reduce sick days

**DOI:** 10.1186/1939-4551-6-15

**Published:** 2013-09-09

**Authors:** Karin D Petersen, Christian Kronborg, Jørgen N Larsen, Ronald Dahl, Dorte Gyrd-Hansen

**Affiliations:** 1 Danish Center for Healthcare Improvements (DCHI), Faculty of Social Sciences and Faculty of Health Sciences, Department of Business and Management, Aalborg University, Fibigerstræde 11, 11-75, 9220 Aalborg Ø, Denmark; 2 Centre of Health Economics Research (COHERE), University of Southern Denmark, Odense, Denmark; 3 Department of Research & Development, ALK-Abelló, Hørsholm, Denmark; 4 Institute for Clinical Medicine, Aarhus Universitet, Brendstrupgårdsvej 100, DK 8200 Aarhus N, Denmark

**Keywords:** Allergic, Rhinitis, Asthma, Health Related Quality of Life, Sick leave, Absenteeism, House dust mite allergy, Immunotherapy, Pollen allergy, Quality adjusted, Life years, Rhino-conjunctivitis, 15D, EQ-5D

## Abstract

**Background:**

One fourth of the adult population in Europe suffer from respiratory allergy. Subcutaneous-allergen-specific-immunotherapy (SCIT) has long-term disease modifying effect on disease specific Health-Related Quality of Life (HRQoL). The purpose of this study was to assess the effect of SCIT on alternative disease outcomes in patients with grass-pollen and/or house dust mite induced allergic rhino-conjunctivitis and/or an asthma diagnosis. Focus was on expressing outcomes in terms of generic quality of life (Quality-Adjusted-Life-Years (QALY)) and reductions in sick days.

**Methods:**

The study was a multi-centre study with prospective follow-up. 248 patients were initiated on SCIT. The disease specific Rhino-conjunctivitis Quality of Life Questionnaire (RQLQ) and two generic (HRQoL) instruments 15D and EQ-5D were used at baseline and at follow-up. The outcome measures included change in; disease severity, RQLQ-scores, number of days with symptoms- and number of sick days per year and finally changes in generic HRQoL and thus, QALY. Disease severity was assessed by specialist doctors; severity of rhino-conjunctivitis was classified according to the Allergic Rhinitis and its Impact on Asthma (ARIA) and asthma severity according to the Global Initiative for Asthma (GINA guideline). The remaining outcome measures were assessed by the patients in questionnaires at baseline and at follow-up. An intension to treat approach was applied. For missing items imputation of sample mean base-line values or follow-up values were used after specified criteria. The effect of SCIT on rhino-conjunctivitis and/or asthma diagnoses was analysed at follow-up using three logistic regression models.

**Results:**

The disease severity showed significantly improved disease control. Mean RQLQ-score was reduced from 3.02 at baseline to 2.00 at follow-up. Average annual days with symptoms were reduced from 189 to 145 days whilst annual sick days were reduced from 3.7 to 1.2 days. The 15D-score increased from 0.83 to 0.86 and the EQ-5D-score from 0.70 to 0.77, which indicated an annual gain per patient of 0.03-0.06 QALY.

**Conclusions:**

Allergic patients suffering from rhino-conjunctivitis alone or rhino-conjunctivitis and asthma experience significantly increased HRQoL and they gain 0.03-0.06 QALY, when treated with SCIT for one year.

**Trial registration:**

The study was registered at ClinicalTrials.gov with the identifier: NCT01486498.

## Background

Respiratory allergic disorders constitute a considerable public health problem in Europe [1,2]. Grass pollen and house dust mites (HDM) are the most common allergens causing allergic rhino-conjunctivitis (RC) and/or asthma. Approximately one fourth of the adult population in Europe suffer from respiratory allergy and the prevalence of grass pollen allergy is on average 18% [3,4].

Interventions against respiratory allergy include symptomatic treatment, allergen avoidance and allergen specific immunotherapy. Subcutaneous allergen specific immunotherapy (SCIT) involves injections with increasing doses of specific allergen vaccine until a maintenance dose is reached, and at regular intervals for 3 years thereafter [5].

Controlled studies have documented that during the pollen season SCIT significantly increases disease specific health related quality of life (HRQoL), reduces symptoms and use of medication [6], and may reduce the risk of developing new allergies and asthma [7,8], however, to our knowledge no studies have investigated the impact of SCIT treatment on generic HRQoL, disease severity classifications and sick days per year in a real life setting.

Relevance of number of sick days and days with allergy symptoms are documented by several studies. One US study documented an annual at-work productivity loss from allergic RC of $ 2.4 - 4.6 billion, with losses aggravated by the use of sedating antihistamines [9]. Another US study showed that 55% of employees reported RC symptoms for an average of 52.5 days, were absent 3.6 days per year due to the condition, and were unproductive 2.3 hours per workday when experiencing symptoms, resulting in a productivity loss of $ 593 per year per employee with allergic RC [10]. A Danish study found that RC patients were absent 2.7 days per year [11], and a Swedish study estimated mean RC absenteeism to 2.3 days per year [12].

Moreover, it is widely acknowledged that the personal burden of illness, as perceived by the allergic RC and/or asthma patient, cannot be fully assessed by traditional measures, such as clinical symptoms, which correlates only moderately with patients’ perceptions and functional capabilities on a daily basis [13,14]. For that reason it is essential to include the patients self-rated HRQoL in evaluations of SCIT interventions.

Recently three self-rated HRQoL instruments were used in parallel to study the baseline HRQoL among 248 Danish allergic RC and/or asthma patients before start of SCIT with grass pollen and/or HDM. The three instruments were the disease specific RC Quality of Life Questionnaire (RQLQ) [15] and two generic instruments EQ-5D and 15D [16,17]. Especially the 15D instrument was able to distinguish HRQoL on a day with allergen exposure from a day without [18-20]. The present study is a follow-up study of this baseline HRQoL study [18].

Economic evaluations frequently measure health outcomes in quality-adjusted life years (QALYs), which is a composite measure combining health-related quality of life (HRQoL) with life expectancy. Typically, generic HRQoL measures are used which provide an index score for various health states (a numerical score that reflects the preferences of the general population regarding a health state compared to other health states). The EQ-5D instrument and the 15D instrument are examples of such generic index-based HRQoL measures.

This study which focuses on both patient related outcomes and specialist doctors’ evaluations in a real life setting made effort in order to capture all possible consequences of SCIT by focussing on multiple dimensions of the public health disease burden. The aim is to analyse and report any effect of SCIT on the following five main outcomes and to calculate a QALY estimate.

1. Disease severity according to international guidelines

2. Disease specific HRQoL (RQLQ)

3. Generic HRQoL (EQ-5D and 15D)

4. Number of days with allergy symptoms per year and

5. Number of sick days per year in patients suffering from RC, with or without asthma, due to grass pollen and/or HDM allergy.

## Methods

The study was a multi-centre study with prospective follow-up. Patients (>16 years) were recruited consecutively from 13 clinics specialised in specific allergy management. The patients were referred to specialist centres by general practitioners for treatment of grass pollen and/or HDM allergy with an indication to initiate SCIT during autumn 2005- spring 2006 and again during autumn 2006- spring 2007, inclusion was not performed during the grass pollen season (GPS). A diagnosis of an allergy to grass pollen and HDM was established with a combination of a relevant clinical history and symptomatology and a skin prick test > 3 mm in diameter and/or presence of allergen specific IgE in serum i.e. spec. IgE > 0.35 kU/L.

Follow-up data was collected on average 14.8 months (SD: 3.2), median: 14.1 month (range: 8.5-25.9) after the first injection date: the first injection date corresponds to our baseline measurements. A total of 248 patients were included (127 with RC only, 121 with RC and asthma). The patients’ mean age was 33.4 (SD 10.8). Treatment was performed using Alutard SQ® *Phleum pratense* and/or Alutard SQ® *Dermatophagoides pteronyssinus*. The maintenance dose was 100.000 SQ units every 6 weeks (ALK-Abello, Horsholm, Denmark).

At follow-up 204 (82.3%) of the patients were accessible in the SCIT program, and had available follow-up data. The reason for dropout was that the patients did not return the follow-up questionnaires, although they were reminded two times.

### Doctor’s questionnaire at baseline and at follow-up (objective/clinical measures)

The specialist doctors provided information about the patients’ general health, disease duration, lung function, and treatment modality and they diagnosed the patients’ disease severity. RC severity was classified according to the Allergic Rhinitis and its Impact on Asthma (ARIA), as intermittent or persistent and as mild or moderate-severe [21], and asthma severity according to the Global Initiative for Asthma (GINA guideline) as intermittent, mild persistent, moderate persistent or severe persistent [22].

### Patients’ questionnaire at baseline and at follow-up

The patients’ questionnaires were filled out by the patients themselves in the specialist doctors’ waiting rooms and collected immediately before SCIT was initiated (baseline), and again after one year (follow-up). The patient questionnaire included the RQLQ, EQ-5D and 15D instruments, questions concerning number of sick days and days with allergy symptoms in the last year as well as socio-demographic items. The reason for using these two generic instruments was to investigate their ability to measure HRQoL in AR and allergic asthma patients, and to increase the robustness of the results by using two different instruments.

RQLQ has 28 questions in seven domains. The overall RQLQ score is the mean of all 28 responses and the domain score is the average score within each domain. Each item has seven levels on an ordinal scale with zero denoting “Not troubled” and six “Extremely troubled”. Patients were asked to fill in the RQLQ items for a typical day with allergic symptoms, instead of last week as prescribed by the standard RQLQ, thereby avoiding distributing questionnaires both in and out of the pollen season.

The 15D questionnaire is a generic self-administrated multi-attribute HRQoL measure assessing health status across 15 dimensions [17]: mobility, vision, hearing, breathing, sleeping, eating, speech, eliminations, usual activities, mental function, discomfort and symptoms, depression, distress, vitality, and sexual activity. Each dimension has five levels, ranging from level one indicating no problems in the dimension to level five signifying extreme problems. The 15 dimensions with the five levels constitute a health state descriptive system which was used to calculate a single index score, the 15D-score. The 15D-score represents the value of the individual respondents overall HRQoL on a scale from zero (equivalent of being dead) to one (equivalent to full health, i.e. no problems on any dimension). The valuation was based on a set of preference weights, elicited from general Danish population [23]. A difference of 0.03 or more in the 15D score is considered clinically important in the sense that it has been found that a person can feel such a difference [24].

The EQ-5D instrument is an alternative health state descriptive system which consists of five dimensions: mobility, self-care, usual activities, pain/discomfort and anxiety/depression. Each item has three levels: no problems, some problems, or extreme problems. Thus, this system represents 243 possible health states. We used the Danish population’s preference weights for these health states to convert the observed health states to a single index score, EQ-5D score, where 1.0 is full health and 0 is dead, and negative scores are considered by the general population as being ‘worse than death’ [23,25].

For the 15D and the EQ-5D items, respondents were asked, both at baseline and at follow-up, to report their general health state on a typical day with allergy symptoms as well as on a typical day without allergy symptoms, instead of their health status today as prescribed by the standard questionnaires. This approach was deemed more appropriate in this case, where symptoms vary from day to day and in and out of season. To supplement this information, respondents were asked to report the number of days in the year that they experience inconvenience from these allergy symptoms, as well as the number of sick days absenteeism (days absent from work) due to allergy symptoms. The latter information was used to estimate productivity loss.

A simple equation of the quality-adjusted life-years (QALY) difference per SCIT treated patient per year was fitted both for the 15D and the EQ-5D scores:

$$\Delta \mathrm{QALY}=\left(a-b\right)\frac{n}{365}+\left(c-b\right)\frac{m}{365}$$

where *a* and *b* is the HRQol score when experiencing allergy symptoms after and before SCIT respectively, and c is the HRQol score on a day without allergy symptoms, *n* is the number of days with allergy symptoms after SCIT, and *m* is the number of days of allergy symptoms avoided after SCIT.

In the intention to treat (ITT) approach the baseline scores were used also (imputed) as follow-up scores for the drop-outs considering the following variables: RC and A diagnoses, HRQoL-scores (EQ-5D, 15D and RQLQ), number of sick days, and days with inconvenience, because of allergy symptoms.

### Pollen counts

As the severity of symptoms of allergic patients is dependent on allergen exposure, any clinical improvement caused by intervention should be evaluated by taking allergen exposure into account [26]. In Denmark pollen counts (available from http://www.polleninfo.org) are collected at two stations, Copenhagen and Viborg. In this study the cumulated pollen count measured in grains/m^3^ during peak season was calculated as the highest sum of 15 consecutive days based on a daily pollen count representing an average between the two pollen stations.

### Statistical analyses

Statistical analyses were performed using STATA 11.0. Continuous variables were described as mean ±1 standard deviation (SD) and categorical variables were described as frequencies. Chi-squared tests were used to test differences on categorical variables. Normal distributed data were analysed using matched paired *t*-test. Where data was not normally distributed Wilcoxon-signed-rank-test was used for ordinal paired variables [27].

For missing items on the HRQoL-scores imputation of sample mean base-line values or follow-up values were used, respectively, if no more than one item was missing in the EQ-5D questionnaire, no more than four items in the 15D questionnaire and no more than seven items in the RQLQ questionnaire. If these criteria were not met the observation was not included in the statistical analyses.

The effect of SCIT on RC and/or asthma diagnoses was analysed at follow-up using three logistic regression models (the difference between the three models was the dependent variable measuring the HRQoL: one model included the RQLQ-scores, one model the EQ-5D-scores and one model the 15D-scores, respectively). Observations, where all dichotomous variables were zero, represented the reference patient for the analysis. For example, the reference patient in the regression model was a male, treated with grass pollen vaccine, and classified as “no or mild hay fever” without asthma. In all analyses a 5% significance level was used.

### Ethical considerations

The study was approved by the Danish Data Agency (2005-41-5534). The scientific ethics committee did not find that the project needed permission from their committee. All patients gave their informed consent prior to inclusion in the study.

The study was registered at ClinicalTrials.gov with the identifier: NCT01486498. Details of the registration can be found at the website of the International Committee of Medical Journal Editors (ICMJE), http://www.icmje.org.

## Results

### The sample

Table 1 presents the socio-economic profile of the vaccinated patients at baseline (N = 248) and at follow-up (N = 204). The table allows for a comparison of persons included at baseline and persons who were successfully followed up and the p-values refer to the drop-out analyses.

**Table 1:** **Socio**-**economic characteristics of patients** (% **or SD**), [] number of observations, P-value refers to the drop-out analyses

**Socio**-**demographic variables**	**Baseline [248] (100%)**	**Follow-up [204] (82.3%)**	**P**-**value**
**Gender**			
Male	126 (50.8)	110 (53.9)	*0.035^a^
**Highest attained education**			
9 years or less school education	31 (12.5)	20 (9.8)	0. 080^a^
Other education or unknown	13 (5.2)	10 (4.9)	
Vocational education and training	30 (12.1)	25 (12.3)	
Short or medium high education	90 (36.3)	77 (37.8)	
University degree	84 (33.9)	72 (35.3)	
**Body mass Index**	24.78 (5.21) [246]	25.00 (5.52) [203]	0.143^b^
Underweight (less than 18.5 kg)	6 (2.4)	5 (2.5)	0.533^a^
Normal weight (18.5 to < =25 kg)	150 (60.5)	120 (58.8)	
Overweight (25 to < = 30 kg)	67 (27.0)	57 (27.9)	
Fat (more than 30 kg)	25 (10.1)	22 (10.8)	
**Household income in** € **in the year 2006 per person in the household**	31,372 (18,061) [214]	32,091 (17,918) [180]	0.181^b^
**Never smoker**	163 (66.0) [247]	134 (66.0) [203]	0.990^a^
**Distance to vaccination place in km**	12.9 (18.0)	12.1 (16.6)	0.154^b^

*Significant difference (p < 0.05) from baseline to follow-up. ^a^Pearsons *x*^2^, ^b^t-test.

There is an equal gender distribution in the baseline sample, but an overweight of patients with higher level of education. More women were lost at follow-up, and there was a tendency for those with lower levels of education to drop out.

At baseline, 169 patients started SCIT with grass pollen immunotherapy only, 25 started SCIT with HDM immunotherapy only, and 54 started treatment with both grass pollen and HDM immunotherapy. 127 of the patients were suffering from RC only and 121 were suffering from RC + asthma. At baseline 15 of the patients were classified as intermittent RC and 233 as persistent RC. Of the 204 patients with follow-up data, 141 had been treated with grass pollen vaccine only, 21 with HDM only and 42 with grass pollen and HDM.

**Figure 1:**
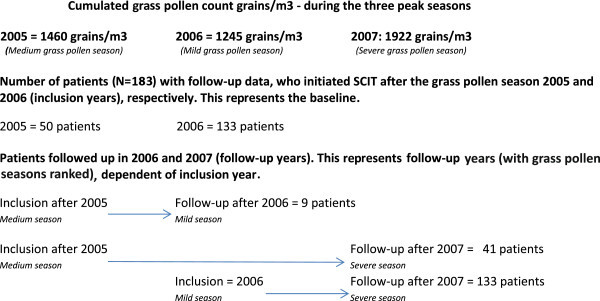
Cumulated grass pollen counts for the three years of the study period and a corresponding flow of the 183 grass pollen allergic patients inclusion- and follow-up year.

Nine (4.9%) of the grass pollen immunotherapy treated patients started SCIT after the grass pollen season 2005 and were followed up after the 2006 season. The main part of the grass pollen immunotherapy treated patients, 133 (72.7%) started SCIT after the 2006 pollen season (cumulated pollen count during peak season: 1245 grains/m^3^ – ranked as a mild season) and were followed up after the 2007 season (cumulated pollen count during peak season: 1922 grains/m^3^ – ranked as a severe season) and the remaining 41 (22.4%) patients started immunotherapy treatment after the 2005 season (cumulated pollen count during peak season: 1460 grains/m^3^ – ranked as a medium season) and were followed up after the 2007 season (see Figure 1).

### Outcomes

Among the 204 patients with available baseline and follow-up data 11 patients had remission of their RC, 30 had remission of their asthma and 3 patients had remission of RC + asthma. Diagnostic criteria for RC were no longer met in 8 patients at follow up.

Table 2 shows the diagnoses and classification of rhino-conjunctivitis according to ARIA and asthma according to the GINA guidelines by treatment modality at baseline and at follow-up with an ITT approach. At follow up, significantly fewer were diagnosed with RC and asthma and the severity of the diseases was diminished significantly (for RC p < 0.013, for asthma p < 0.001). The patients experienced on average 44 fewer days with inconvenience a year (p < 0.001) and 2.5 fewer sick days a year at follow-up (p < 0.001). The HRQoL measures (RQLQ, 15D and EQ-5D) on days with allergen exposure were all significantly improved (p < 0.001).

**Table 2:** **Diagnoses and classification of rhino**-**conjunctivitis (RC) and asthma by treatment modality at baseline** (**B**) **and at follow**-**up** (**F**) **with an intension to treat approach N** = **248**

	**Grass pollen only N = 169**		**House dust mites(HDM)only N = 25**		**Grass and HDM mites N = 54**		**Full sample N = 248**		
	**B**	**F**	**B**	**F**	**B**	**F**	**B**	**F**	**P-value**
**RC diagnosis, ± classification**									
RC diagnosis	169	166	25	23	54	48	248	237	
No RC diagnosis	0	3	0	2	0	6	0	11	0,013 ^a^
Intermittent RC	7	57	4	8	4	18	15	83	
Persistent RC	162	109	21	15	50	30	233	154	
**Asthma diagnosis**									
Asthma diagnosis	81	70	12	8	28	24	121	102	< 0.001^a^
No asthma diagnosis	88	99	13	17	26	30	127	146	
Intermittent	29	27	6	3	7	8	42	38	
Mild Persistent	21	22	1	1	6	3	28	26	
Moderate Persistent	28	18	4	3	11	12	43	33	
Severe Persistent	3	3	1	1	4	1	8	5	
**Days with inconvenience last year mean(SD)**	160.2 (105.1)	119.6 (118.5)	270.9 (102.8)	191.1 (137.6)	247.2 (134.0)	185.3 (142.8)	189.3 (119.9)	141.1 (123.4)	< 0.001^b^
**Sick**-**days last year mean(SD),‘median,min-max days’**	3.49 (17.78) ´0, 0-220`	0.88 (3.04)´ 0, 0–22.5`	6.75 (13.72) ´0, 0-55`	4.00 (12.51) ´0, 0-55`	2.87 (6.05) ´0, 0-30`	0,87 (2.30) ´0, 0-10`	3.68 (15.58) ´0, 0-220`	1.19 (4.82) ´0, 0-55`	< 0.001^b^
**HRQoL-scores mean(SD)**									
RQLQ*	3.12 (0.68)	2.01 (0.78)	3.21 (0.66)	2.34 (0.76)	2.61 (0.79)	1.83 (0,85)	3.02 (0.74)	2.00 (0.80)	< 0.001^b^
EQ-5D-score on days with no allergen exposure	0.98(0.06)	0.98 (0.07)	0.93 (0.13)	0.97 (0.08)	0.98 (0.05)	0.99 (0.04)	0.98 (0.07)	0.98 (0.07)	0.975
EQ-5D-score on days with allergen exposure	0.70 (0.18)	0.78 (0.15)	0.60 (0.30)	0.67 (0.27)	0.72 (0.18)	0.80 (0.15)	0.70 (0.20)	0.77 (0.17)	< 0.001^b^
15D-score on days with no allergen exposure	0.98 (0.03)	0.98 (0.04)	0.97 (0.05)	0.97 (0.05)	0.98 (0.03)	0.99 (0.02)	0.98 (0.03)	0.98 (0.04)	0.180
15D-score on days with allergen exposure	0.83 (0.08)	0.86 (0.08)	0.79 (0.09)	0.81 (0.11)	0.83 (0.08)	0.88 (0.07)	0.83 (0.08)	0.86 (0.08)	< 0.001^b^

*Rhino-conjunctivitis Quality of Life Questionnaire, ^a^Significant difference (p < 0.05) from baseline to follow-up Fishers Exact *x*^2^ test, ^b^significant difference (p < 0.05) from baseline to follow-up Wilcoxon signed-rank test.

An estimate (based on 15D scores) of the QALY´s gained per SCIT patient per year would be equivalent to (0.03*145/365) + (0.98-0.83)* 44/365 = 0.03 QALYs gained (where 0.98 and 0.83 are the 15D base-line scores on a day without allergy and with allergy, 44 (189–145) is the number of days with allergy avoided by SCIT, 0.03 (0.86-0.83) is the gain in HRQoL on a day with allergy after SCIT and 145 are the number of days with allergy after SCIT) see Table 2. Using EQ-5D scores the annual QALY gain would be 0.06.

Table 3 presents three logistic regression models with positive change in allergic RC diagnosis from baseline to follow-up as dependent variable (= 1 loss of diagnosis, which means cure); = 0 if no change of diagnosis). The three models include each the relevant HRQoL baseline measures RQLQ, EQ-5D and 15D, respectively, as independent variable along with gender, age at inclusion, BMI, treatment modality and clinic setting (public hospital = 0 or private clinic = 1). The effectiveness of SCIT in the treatment of allergic RC was positively associated with increasing age (the RQLQ p = 0.018; the 15D p = 0.020; the EQ-5D p = 0.018), and treatment with two allergens, i.e. grass pollen and HDM (the RQLQ p = 0.091; the 15D p = 0.026; the EQ-5D p = 0.032). Baseline HRQoL-scores, gender, BMI and treatment framework were not associated with the likelihood of a loss of the RC diagnosis and irrespective of HRQL instrument used.

**Table 3:** **Three models of predicting loss of the diagnoses of rhino-conjunctivitis (RC)** (=**1**) **at follow**-**up**

	Model one With RQLQ** ***in the model***			***Model two With 15D***-***score in the model***			***Model three With EQ***-***5D***-***score in the model***		
	**Coefficient**	**Std**. **Err**	**P**-**value**	**Coefficient**	**Std**. **Err**	**P**-**value**	**Coefficient**	**Std**. **Err**	**P**-**value**
Gender – male	−0.900	0.754	0.232	−0.733	0.725	0.312	−0.696	0.722	0.335
Age at inclusion	0.069	0.029	0.018*	0.067	0.029	0.020*	0.070	0.029	0.018*
Body mass index	−0.126	0.093	0.175	−0.123	0.100	0.218	−0.122	0.102	0.233
Vaccination modality House dust mites only	1.432	0.992	0.149	1.421	0.988	0.150	1.464	0.981	0.136
Grass pollen and house dust mites	1.367	0.808	0.091	1.697	0.762	0.026*	1.638	0.766	0.032*
HRQoL^^-scores at baseline	−0.683	0.500	0.172	2.989	4.469	0.504	1.905	2.186	0.383
Vaccination place – private clinic	1.033	1.193	0.387	0.890	1.144	0.437	0.940	1.147	0.412
Constant	−1.842	2.750	0.503	−6.343	3.941	0.108	−5.352	2.675	0.045
Number of observations	245			244			241		
LR chi^2^	17.76			16.21			16.96		
Prob > chi^2^	0.0131			0.0232			0.0177		
Pseudo R^2^	0.1979			0.1808			0.1897		

*p < 0.

## Discussion

According to this study, it seems that patients treated with SCIT for allergic RC ± asthma experience reduced disease severity, decreased days with allergy symptoms, increased disease specific and generic HRQoL and decreased number of sick days, which are all highly relevant patient related outcomes and of general importance to the public health issues of allergic diseases.

The results indicate that SCIT provides improvements in general quality of life. A conservative estimate (based on 15D scores) of the QALY´s gained by SCIT was equivalent to 0.03 QALYs gained per SCIT treated patient per year. Using EQ-5D scores the annual QALY gain would be 0.06. Patients treated for both HDM and grass pollen allergy achieved the greatest HRQoL-gains measured on the two generic instruments.

Different HRQol instruments produced different measures and results. In this study three different but very relevant instruments were applied to elucidate the HRQoL scores. As observed in other studies, the ED-5D detected a greater difference in HRQol-score as compared to the 15D instrument [19,20,28,29].

The strength of applying generic instruments for the assessment of HRQoL is that the total scores represent a weighted average across dimensions, with the relative weighting being based on the publics’ preferences. Such evaluations represent a useful tool for decision makers and may represent a valuable contribution to the optimising of resource allocation across different patient groups [30].

An ITT approach was applied, where the self-reported baseline HRQoL-scores, number of sick days and days of inconvenience were assumed unchanged at follow-up for the 44 patients for whom follow-up data was not available. The reported SCIT effect is likely to be a conservative estimate, as some of the 44 study drop-outs did complete the up-dosing phase and thereby have achieved much of the gain by SCIT [31].

Patients filled in the questionnaires as they recalled a typical day with allergen exposure and a typical day without allergen exposure, which may have produced some degree of recall bias. Although the exact correlation between grass pollen exposure and AR and/or asthma severity is unknown, the burden of AR and asthma could be influenced by the GPS [26].

In this study the grass pollen allergic part of the included patients constituted the main part of patients. Since the main part of the patients treated with grass pollen vaccine (72.7%) started SCIT after the 2006 pollen season, which was a relatively mild season it is unlikely that the effect size is exaggerated by the GPS or regression toward the mean.

Most of the patients in this study had their follow-up evaluation after the 2007 pollen season, and this pollen season was clearly higher in grass pollen count (ranked as a severe season) compared to the two previous seasons, where the patients had their baseline assessment. The clinical improvements observed in this study therefore represent conservative estimates and any bias, e.g. regression to the mean, is likely to reflect an underestimation of the effect of SCIT.

The effectiveness of SCIT in the treatment of allergic RC was positively associated with treatment with two allergens, dual grass pollen-HDM. This is because the period of the perennial symptoms in those with HDM allergy in addition to a seasonal allergy achieves a much longer time with improved HRQoL compared to monosensitized persons, which translate into a higher QALY.

One weakness of the study was the missing control group and a possible placebo effect. It is however difficult in a real life setting to have a matched group for correct comparison and this study report the findings before and after the intervention. The gains in RQLQ scores due to SCIT were in line with other studies [6,32,33]. Earlier studies have typically focused on medicine scores, symptom scores and sick days [32]. Because of the inclusion of various outcome measures, based on objective observations by allergy specialists as well as patients’ subjective experiences, this study provides evidence of the positive effect of SCIT.

Women were more inclined to drop out of the study, and there was also a tendency for those with lower levels of education to drop out. The absolute difference in numbers (see Table 1) was small suggesting that the impact on results is limited. From an earlier study it was found that allergic patients with a university degree as well as younger patients were more likely to commence a SCIT treatment [19,31] suggesting that motivations in this sub-groups are higher.

An important effect of SCIT was a reduction in sick days from work averaging 2.5 days and a reduction of 44 days of inconvenience per annum in the mixed grass pollen and/or HDM allergic cohort. This effect is likely to decrease the burden of disease significantly for patients, employers and for society. A conservative estimate on gained sick days by excluding an outlier having 220 days of sick leave at baseline, still shows a significant and important gain of average 1.6 days annual sick days per SCIT treated patient and is in agreement with an earlier study in Denmark from 2005, where the gain in avoided sick days was found to be 2.1 days per year [31]. It is, however, important to stress that in this study only absenteeism was included and no presenteeism was incorporated in the analyses, which makes the estimate conservative since the productivity losses caused by being at work but producing less were not included. Presenteeism is the situation where a person will attend work while sick, which may be a disadvantage to the health and work performance. Other studies have shown presenteeism to constitute the greater part of productivity loss for the society [10].

## Conclusions

This study shows that treatment with SCIT of allergic patients suffering from RC and/or asthma is followed by a marked improvement in all primary outcomes: diagnosis severity is reduced, HRQoL-scores, number of sick days and days with inconvenience per year due to allergy. All these outcome measures were captured in the QALY measure, which estimated a QALY gain in the order of 0.03-0.06 QALY´s.

## Abbreviations

15D: 15-dimensional instrument for measuring HRQL; EQ-5D: Euro-Qol with 5 dimensions; AR: Allergic Rhinitis/rhino-conjunctivitis; RC: Rhino-conjunctivitis; GPS: Grass pollen season; HRQoL: Health related quality of life; RQLQ: Rhino-conjunctivitis quality of life questionnaire; QALY: Quality adjusted life years; ARIA: Allergic rhinitis and its impact on asthma; GINA: Global initiative for asthma; HDM: House dust mite.; ITT: Intention to treat; BMI: Body mass index; SCIT: Subcutaneous allergen specific immunotherapy.

## Competing interests

Funding for this project was provided by a consortium consisting of the University of Southern Denmark, ALK A/S, and the Danish Ministry of Science, Technology and Innovation in the form of an industrial PhD study for Karin Dam Petersen (Project number 61493-F). The parties entered a contractual agreement within the Danish Agency for Science, Technology and Innovation ensuring that no competing interests were to influence the conduct of the research. Jørgen Nedergaard Larsen (JNL) is an employee of ALK A/S. Karin Dam Petersen (KDP) has received honorarium from ALK A/S as a speaker at the Symposium of Specific Allergy SOSA conference in 2008 in Copenhagen. The three remaining authors Dorthe Gyrd-Hansen (DGH), Christian Kronborg (CK) and Ronald Dahl (RD) have no conflict of interest in relation to the research described in the enclosed manuscript.

The study drugs, Alutard SQ® Phleum pratense and/or Alutard SQ® Dermatophagoides pteronyssinus, are mentioned in the methods section of the manuscript, and they are manufactured and marketed in Denmark by ALK A/S.

## Authors’ contribution

KDP, CK, JNL, RD and DGH conceived the study, carried out the statistical analyses and drafted the manuscript. KDP, DGH and CK were involved with acquisition of the data and the data management. All authors participated in the design of the study, helped draft the manuscript, helped improve the analyses and interpret the results. The work has not been published before and is not being considered for publication elsewhere. All authors have approved the final manuscript.
